# Vitamin D Intake from Foods, Supplements, and Food Sources: Findings from the Hispanic Community Health Study/Study of Latinos

**DOI:** 10.1016/j.tjnut.2025.09.002

**Published:** 2025-09-10

**Authors:** Izabelle Mendez, Nicole Farmer, Jacqueline J Pujol, Juliana T Camargo, Amanda S Hinerman, Erik J Rodriquez, Anna M Nápoles, Martha L Daviglus, Amber Pirzada, Christina Cordero, Daniela Sotres-Alvarez, Eliseo J Pérez-Stable

**Affiliations:** 1 Division of Intramural Research, National Institute on Minority Health and Health Disparities, National Institute of Health, Bethesda, MD, United States; 2 Division of Intramural Research, National Heart, Lung and Blood Institute, National Institutes of Health, Bethesda, MD, United States; 3 Translational Biobehavioral and Health Disparities Branch, National Institutes of Health, Clinical Center, Bethesda, MD, United States; 4 Department of Biostatistics, Gillings School of Global Public Health, University of North Carolina at Chapel Hill, Chapel Hill, NC, United States; 5 Institute of Minority Health Research, University of Illinois Chicago College of Medicine, Chicago, IL, United States; 6 Department of Psychology, University of Miami, Miami, FL, United States

**Keywords:** vitamin D, Hispanic or Latino, dietary supplements, diet, micronutrients

## Abstract

**Background:**

Hispanic/Latino adults have a higher prevalence of vitamin D deficiency than other adults.

**Objectives:**

The objective of this was to evaluate the long-term mean daily intake of vitamin D (food and supplements), vitamin D-contributing food sources, and adherence to the Estimated Average Requirement (EAR) for vitamin D among Hispanic/Latino adults.

**Methods:**

The Hispanic Community Health Study/Study of Latinos is a population-based cohort study of 18–74-y-olds from 4 cities and 6 heritage groups: Central American, Cuban, Dominican, Mexican, Puerto Rican, and South American (visit 1: 2008–2011). We included participants who completed 2 24-h dietary recalls and food propensity questionnaire (*n* = 13,340). Vitamin D sources included fish, eggs, dairy, plant-based milk alternatives, citrus juice, ready-to-eat cereals, organ meats, yogurt, margarine, cheese, and vitamin D supplements. Total usual nutrient intake of vitamin D from food and dietary supplements, as well as servings of vitamin D-contributing food sources, were estimated.

**Results:**

Dietary supplement users (*n* = 3459) were more likely to have health insurance (56.7% compared with 48.8%, *P* < 0.001) and an annual household income of >$75,000 (9.3% compared with 4.9%, *P* < 0.001) than nonusers (*n* = 9881). Usual vitamin D intake from food alone was similar for dietary supplement users and nonusers (mean daily intake 5.14 μg compared with 5.06 μg). Dietary supplement users had a higher daily mean intake (17.41 μg compared with 5.06 μg) and a lower proportion being below the EAR (32.3% compared with 95.2%) than nonusers. Fish products (0.655 oz/d) and dairy milk (0.699 cup/d) were the most consumed vitamin D food sources.

**Conclusions:**

Regardless of dietary supplement use, mean vitamin D intake from usual food consumption alone was insufficient to meet the EAR. Supplementation was associated with a greater likelihood of meeting the EAR. The information on vitamin D supplementation and variation in specific food sources can inform the development of tailored interventions for Hispanic/Latino populations at greater risk of vitamin D deficiency.

## Introduction

Vitamin D is a fat-soluble vitamin that plays a critical role in maintaining skeletal integrity, electrolyte reabsorption, and immune system regulation [1]. Based on an NHANES study, Hispanic/Latino adults have been found to have lower serum 25-hydroxyvitamin D concentrations than White adults [2] and a higher prevalence of vitamin D deficiency (63% compared with 30%) [3]. Moreover, Hispanic/Latino adults have the second highest prevalence rate of vitamin D deficiency across all major United States population groups [3]. Given the evidence linking vitamin D deficiency to increased risk of fractures and aging-related functional limitations in adults, reducing vitamin D deficiency among Hispanic/Latino adults is an important prevention strategy to reduce health disparities [[3], [4], [5], [6]].

Although sunlight is the primary source of vitamin D, it is insufficient to maintain healthy concentrations due to seasonal, cultural, behavioral factors, clothing practices associated with outdoor work or sun safety, elevated melanin pigmentation, and sunblock application, can impede vitamin D synthesis [7,8]. Therefore, dietary vitamin D intake is essential in achieving adequate vitamin D concentrations. The Estimated Average Requirement (EAR) or daily recommended vitamin D intake concentration for which 50% of healthy adults aged 19–70 y would meet their vitamin D needs is 400 International Units (IUs) or 10 μg [9]. Vitamin D is found in natural food sources such as fatty fish (e.g., salmon, sardines, and tuna), fish liver oils, egg yolks, organ meats, and fortified foods (e.g., ready-to-eat cereals, orange juice, dairy, and plant-based milk alternatives) [7]. Foods that contribute most to dietary vitamin D intake vary across countries and populations, based on dietary patterns and culture [10,11]. In the United States, milk, meat, and fish are the leading food sources of vitamin D [10,12,13]. Dietary supplements containing vitamin D have become more common and frequently consumed [14]. Vitamin D can be found in multivitamin/multimineral formulations as well as a single supplement in a range of dosage concentrations, including 1000–5000 IU of vitamin D3 and ≤50,000 IU of vitamin D2 [14]. Although inadequate intake of vitamin D from foods and/or supplements is common, certain medical conditions may also contribute to vitamin D deficiency (e.g., malabsorption due to celiac disease) [15].

A United States study examining intakes of vitamin D from food and supplement sources found that the daily intake of vitamin D differed by race and/or ethnicity, with Mexican American individuals having the lowest [16]. Within vitamin D food sources, they found that fortified milk and ready-to-eat cereal, combined, accounted for a large portion of the daily vitamin D intake in the overall study population [16]. However, the study’s estimates were based on data collected in NHANES 1999–2000, which focused mostly on one Hispanic/Latino subpopulation: Mexican Americans. Another study examined the usual vitamin D intake among various Hispanic/Latino heritages in the United States; however, they did not include dietary supplement intake [17]. Without the inclusion of nutrients obtained from dietary supplements, the prevalence of individuals below recommended dietary reference intakes may be overestimated [18]. Total usual nutrient intake ideally captures nutrients obtained from food (fortified and unfortified) and dietary supplements.

A recent study evaluated the total usual nutrient intake (food plus supplements) and major food group sources using DRI values (percent below EAR) for vitamin D; however, it was among Canadian individuals and did not include Hispanic/Latino adults [19]. United States studies examining the total usual intake of vitamin D and major vitamin D food sources among Hispanic/Latino adults by heritage are lacking. Furthermore, identification of major contributors to dietary vitamin D intake among Hispanic/Latino adults can inform dietary strategies and recommendations based on food preferences and behaviors [20]. Therefore, our study aimed to provide data on vitamin D dietary supplement intake and vitamin D food sources consumed, to assess their respective contributions to overall vitamin D intake. Information on variation across heritage groups in vitamin D intake from specific food sources and vitamin D supplementation can be used to develop tailored interventions and public health and nutrition messaging for Hispanic/Latino heritage groups at greater risk of vitamin D deficiency. We hypothesize that vitamin D intake concentrations and the likelihood of meeting the EAR for vitamin D will vary by heritage and that dietary supplement users will be more likely to meet the EAR compared with nonusers.

## Methods

The Hispanic Community Health Study/Study of Latinos (HCHS/SOL) is a population-based cohort study of Hispanic/Latino adults living in 4 regions of the United States: Bronx, NY; Chicago, IL; Miami, FL; and San Diego, CA. HCHS/SOL recruited 16,415 adults (18–74 y) between the years 2008 and 2011. By heritage, the HCHS/SOL cohort includes participants who self-identify as Mexican (*n* = 6,472), Puerto Rican (*n* = 2,728), Cuban (*n* = 2,348), Dominican (*n* = 1,473), Central American (*n* = 1,732), and South American (*n* = 1,072). The overall study design has been previously described [21]. All participants with vitamin D food and dietary supplement intake information from completed 24-h food and supplement recalls (*n* = 15,973) in Visit 1 (years: 2008–2011) and food propensity questionnaires (FPQs) in the 1-y follow-up (year: 2012) were included in this study; those with missing dietary (*n* = 991) or supplement data (*n* = 1881) were excluded. As recommended by HCHS/SOL Dietary Data Guidelines, recalls deemed unreliable by interviewer (*n* = 614) and those with extreme energy intake (defined as below the sex-specific first percentile or > 99th percentile) (*n* = 165) were also excluded [17]. The final analytic sample was *n* = 13,340.

### Vitamin D dietary data collection

Daily vitamin D dietary intake was assessed by 2 nonconsecutive 24-h recalls that included both the food and dietary supplement recalls and a 30-d recall for dietary supplements at Visit 1 (years: 2008 to 2011). The Nutrition Data System for Research software’s Dietary Supplement Assessment Module was used to collect and analyze the 24-h and 30-d recall data. This software allowed for the collection of 24-h and 30-d intake of all dietary foods (including eating occasions, mealtime, cooking methods, seasonings, and brand names) and supplements (including multivitamin or singular). Detailed information on the consumption frequency, serving amount, and dose (e.g., μg) was also collected. Vitamin D dietary intake was reported in micrograms per day [17]. In this analysis, dietary supplement users and nonusers were defined by the mean vitamin D supplement intake from the 30-d dietary supplement recalls.

Food source consumption was also assessed using the FPQ versions A and B, which were administered at the follow-up call 1 year after visit 1 (year: 2012). The FPQ asked participants to report frequencies of foods eaten in the previous year. The FPQ version A was shortened to reduce participant burden and eliminate food sources that were poorly correlated between 24-h recalls and the FPQ (*r* < 0.5 and *P*-monotone trend > 0.05). After all exclusions were applied, the FPQ version B was reduced from 139 food items and 228 individual questions to 115 food items and 137 individual questions, which represented a 40% reduction in the number of items. There was no dietary information collected at Visit 2 (years: 2014–2017), and therefore, we are unable to assess trends over time. Serum 25-hydroxyvitamin D concentrations, which assess vitamin D status, were not measured and could not be incorporated in this study.

### Total usual dietary intake of vitamin D (food sources and dietary supplements)

Usual total daily vitamin D intake distributions from food sources were estimated using the National Cancer Institute (NCI) statistical method described elsewhere in detail [17,22]. To incorporate vitamin D dietary supplements into the total usual daily intake, the “shrink-then-add” method was used as discussed in the Statistical Analysis section below and described elsewhere in detail [18].

### Vitamin D food sources

Vitamin D food sources were categorized as follows: *1*) fish, *2*) eggs and egg substitutes (e.g., egg analogs, imitating whole eggs or a component of the egg, such as egg whites), *3*) milk (including dairy and plant-based alternatives), *4*) citrus juice (including sweetened or unsweetened orange, grapefruit, tangerine), *5*) organ meats, *6*) cheese, *7*) yogurt, *8*) margarine, and *9*) ready-to-eat cereals (Supplemental Table 1). The usual serving intakes of vitamin D food sources were estimated by applying the NCI method. The FPQ versions A and B were merged and used as a covariate to improve estimates of usual intake for episodically consumed foods (foods not consumed on a daily basis by entire population and commonly reported as zero on a particular day) [23] The sum of the frequencies of several individual FPQ items was used to represent the frequency of consumption of a food source containing them [23]. Serving sizes have been assigned to each Nutrition Data System for Research food based on the recommendations made by the 2000 Dietary Guidelines for Americans (fish: one serving = 1 oz; organ meat: one serving = 1 oz; eggs/egg substitutes: one serving = 1 large egg; milk: one serving = 1 cup fluid milk; citrus juice: one serving = 4 fluid oz; ready-to-eat cereal: one serving = 1 oz; margarine: one serving = 1 tsp; yogurt: one serving = 1 cup; cheese: one serving = 1 1⁄2 oz natural, 2 oz processed, 2 cups cottage, 3 cups dry curd, 1⁄2 cup ricotta, or 2 oz cheese spread).

### EAR

The EAR was used to assess if participants’ mean vitamin D intake was within the recommended dietary reference intake concentrations of adequacy [24]. The cut-point method assesses the nutrient adequacy of groups below the EAR. The threshold used for defining inadequate vitamin D intake was an EAR of < 400 daily IU (10 μg). We considered groups to be at risk of inadequate intake of vitamin D if <50% of individuals met the EAR value [24].

### Other covariates of interest

Covariates included age (18–29, 30–49, 50–69, or 70–74 y), sex (female or male), educational attainment (<high school/GED, high school/GED, or greater than high school/GED), annual household income (<$10,000, $10,001–$20,000, $20,001–$40,000, $40,001–$75,000, or > $75,000), marital status (single, married, or separated/divorced), current insurance coverage (yes/no), acculturation (Short Acculturation Scale in Hispanics [SASH] language subscale score) [25]. SASH Language score was determined by taking the mean of 4 items from the Short Acculturation Scale for Hispanics, which assessed language use in reading, speaking at home, thinking, and speaking with friends[25]. Responses range from 1 to 5, with higher scores indicating better English language orientation. Place of birth/duration of United States residence (born in 50 United States states not including United States territories; born outside 50 states and duration of United States residence ≥10 y; born outside 50 states and duration of US residence <10 y), occupation (nonskilled worker, service worker, skilled worker, or professional/technical/other office worker), employment status [full-time (>35 hours/wk), part-time (≤35 h/wk), unemployed, or retired], and body mass index [BMI: underweight (<18.5 kg/m^2^), normal (18.5–24.9 kg/m^2^), overweight (25.029.9 kg/m^2^), obese (30+ kg/m^2^)]. Hispanic/Latino heritage [Central American, Cuban, Dominican, Mexican, Puerto Rican, South American, and other/missing (multi/mixed ethnic backgrounds)].

Self-reported chronic conditions included hypertension, angina, heart failure, peripheral arterial disease, diabetes, chronic kidney disease, liver problems (e.g., hepatitis, cirrhosis), and cancer. The number of chronic conditions (none, 1, 2, or more) was also assessed. Other variables included current smoking status (yes, no), physical activity level (minutes/week: high, moderate, or low), and diet quality, assessed by the Alternate Healthy Eating Index-2010 (AHEI-2010) score. The AHEI-2010 consists of 11 components—whole grains, fruit, vegetables, nuts and legumes, omega-3 fatty acids, PUFAs, sugar-sweetened beverages and fruit juice, red and processed meats, trans fats, sodium, and alcohol—scored from 0 to 10 based on intake. Total scores range from 0 to 110, with higher scores indicating better dietary quality [26,27].

### Statistical analysis

Demographic and health-related characteristics were compared using t-tests for continuous variables and chi-squared tests for categorical variables.

Usual dietary intake distributions for vitamin D and vitamin D food sources were estimated using the NCI method, a 2-part mixed-effects modeling approach that uses repeated 24-h dietary recalls to estimate the probability of consumption while enabling adjustment for covariates and corrections for measurement error [17]. To incorporate supplement consumption into the total usual dietary intake distribution for vitamin D and to improve estimation accuracy, we used the “shrink-then-add” method as described in further detail elsewhere [18].

For the usual dietary intake estimation, the dietary measurement error models were fit for the 24-h recalls of vitamin D and vitamin D food sources, adjusting for sex, age, Hispanic/Latino heritage, field center, weekend consumption, if intake amount was self-reported to be more or less than usual, and sequence [17]. To improve estimates for episodically consumed foods, models for the vitamin D food sources were additionally adjusted for self-reported consumption frequency from the FPQ.

The dietary measurement error models were fit using the SAS macro MIXTRAN, version 2.21, available from the NCI Division of Cancer Prevention website on Software for Measurement Error in Nutrition Research [28], with specification of a one-part nonlinear mixed model for vitamin D and 2-part nonlinear mixed model for vitamin D food sources. The 2-part nonlinear mixed model allowed for correlated random effects between the amount-part model and the consumption-part model, except for nondairy milk alternatives, where uncorrelated random effects were used to facilitate convergence during model fitting. The usual daily intake distributions were estimated using the Monte Carlo approach implemented in the SAS macro DISTRIB, version 2.2, available from the NCI Division of Cancer Prevention website on Software for Measurement Error in Nutrition Research [28]. For consideration of vitamin D dietary supplementation, the final step of the "shrink-then-add" approach involved adding the vitamin D supplement values to the appropriate intake values from the previous Monte Carlo step.

The mean usual daily intake of vitamin D with and without supplementation, and mean usual daily intake of vitamin D food sources, were calculated by heritage and compared using t-tests. Daily mean supplement intake alone was also compared by heritage using t-tests. Percentage of intake below the EAR cut point was calculated and compared by heritage and supplement use status. The mean relative contribution of dietary supplements to total daily usual intake was calculated by dividing the intake from supplements by the total usual vitamin D intake from both food and supplement sources, individually for dietary supplement users and averaged to compare by heritage.

Bootstrap sampling weights were used to estimate SEs and hypothesis tests for group comparisons. All calculations were performed using complex survey procedures in SAS, version 9.4, to account for HCHS/SOL’s complex survey design.

## Results

### Study population

Sample demographics and other characteristics overall (*n* = 13,340), by vitamin D dietary supplement use, are reported in Table 1. Overall, the majority (79.2%) of the sample was born outside of 50 United States States/DC and had a relatively low mean acculturation language score (2.1). Additionally, most of the sample did not consume vitamin D dietary supplements in the last 30 d (74.1% of nondietary vitamin D supplement users compared with 25.9% of vitamin D supplement users). On mean, vitamin D dietary supplement consumers were older (46.5 compared with 40.5 y old) than those who did not consume supplements. Dietary supplement users had significantly larger proportions of 50–69 year olds (37.6% compared with 25.6%; *P* < 0.001), Puerto Rican adults (16.9% compared with 14.1%; *P* = 0.002), Mexican adults (38.7% compared with 35.9%; *P* = 0.002), married or living with a partner status (53.4% compared with 49.9%; *P* <0.001), income >$75,000 (9.3% compared with 4.9%; *P* < 0.001), those living in San Diego (28.3% compared with 22.7%; *P* = 0.004) and health insurance (56.7% compared with 48.8%; *P* < 0.001) as well as more years lived in the United States (24.7 compared with 18.8; *P* < 0.001), higher mean score on language acculturation (2.2 compared with 2.1, *P* = 0.0023) and higher mean diet quality scores (49.5 compared with 47.1; *P* < 0.001) than nonusers, respectively.

**TABLE 1:** Characteristics at Visit 1, stratified by vitamin D dietary supplement use, Hispanic Community Health Study/Study of Latinos, 2008–2011^1^.

	Total sample	Supplement users	Nonsupplement users	Chi- (or T-) *P*-value^2^
Total, *n* (%)	13,340	3459 (25.9)	9881 (74.1)	
Age (y), mean (SE)	42.0 (0.26)	46.5 (0.47)	40.5 (0.27)	<0.001
Age (y), *n* (%)				<0.001
18–29	1957 (24.7)	264 (14.3)	1693 (27.9)	
30–49	5380 (43.6)	1263 (42.6)	4117 (43.8)	
50–69	5593 (28.4)	1774 (37.6)	3819 (25.6)	
70–74	405 (3.3)	157 (5.5)	248 (2.6)	
Sex, *n* (%)				<0.001
Female	8147 (53.5)	2312 (58.5)	5835 (52.0)	
Male	5193 (46.5)	1147 (41.5)	4046 (48.0)	
Hispanic/Latino heritage, *n* (%)				0.0023
Central American	1423 (7.5)	357 (7.2)	1066 (7.6)	
Cuban	2064 (22.1)	384 (17.9)	1680 (23.4)	
Dominican	1182 (9.8)	283 (9.2)	899 (9.9)	
Mexican	5242 (36.6)	1457 (38.7)	3785 (35.9)	
Puerto Rican	2130 (14.8)	629 (16.9)	1501 (14.1)	
South American	897 (5.2)	248 (5.8)	649 (5.0)	
Other/missing	402 (4.1)	101 (4.3)	301 (4.0)	
Field center, *n* (%)				0.0040
Bronx	3159 (23.4)	828 (23.3)	2331 (23.4)	
Chicago	3359 (25.3)	873 (24.4)	2486 (25.6)	
Miami	3542 (27.2)	737 (24.0)	2805 (28.2)	
San Diego	3280 (24.1)	1021 (28.3)	2259 (22.7)	
Marital status, *n* (%)				<0.001
Single	3448 (31.9)	754 (25.3)	2694 (34.0)	
Married or living with a partner	7060 (50.8)	1823 (53.4)	5237 (49.9)	
Separated, divorced, or widow(e)	2807 (17.3)	878 (21.2)	1929 (16.1)	
Education, *n* (%)				<0.001
<high school/GED	4958 (31.5)	1219 (28.4)	3739 (32.5)	
High school/GED	3379 (27.7)	773 (23.8)	2606 (28.9)	
> high school/GED or some college	1718 (12.6)	473 (13.4)	1245 (12.4)	
University or college	3261 (28.1)	989 (34.5)	2272 (26.2)	
Employment status, *n* (%)				<0.001
Retired and not currently employed (or missing on employment)	1279 (8.7)	439 (12.9)	840 (7.4)	
Not retired (or missing on retirement) and not currently employed	5202 (40.7)	1154 (36.0)	4048 (42.2)	
Employed part-time (≤35 h/wk)	2232 (16.7)	611 (17.0)	1621 (16.7)	
Employed full-time (>35 h/wk)	4485 (33.9)	1210 (34.1)	3275 (33.8)	
Occupation, *n* (%)				<0.001
Nonskilled worker	2015 (26.6)	514 (23.6)	1501 (27.5)	
Service worker	1329 (19.2)	340 (18.2)	989 (19.5)	
Skilled worker	1502 (22.7)	429 (24.3)	1073 (22.3)	
Professional/technical, etc.	832 (14.2)	272 (18.3)	560 (12.8)	
Other occupation	1063 (17.4)	276 (15.5)	787 (17.9)	
Annual household income^3^, *n* (%)				<0.001
<$10,000	1850 (14.5)	449 (13.7)	1401 (14.7)	
$10,001–$20,000	3978 (31.4)	938 (26.6)	3040 (33.0)	
$20,001–$40,000	4208 (33.6)	1129 (32.8)	3079 (33.9)	
$40,001–$75,000	1667 (14.5)	526 (17.5)	1141 (13.5)	
>$75,000	535 (6.0)	184 (9.3)	351 (4.9)	
United States born, *n* (%)				0.5690
Not born in 50 United States States/DC	11,174 (79.2)	2891 (78.7)	8283 (79.4)	
Born in 50 United States States/DC only	2155 (20.8)	567 (21.3)	1588 (20.6)	
Insurance coverage, *n* (%)				<0.001
No current health insurance	6494 (49.3)	1517 (43.3)	4977 (51.2)	
Currently have health insurance	6686 (50.7)	1899 (56.7)	4,787 (48.8)	
Years lived in the United States, mean (SE)	20.2 (0.35)	24.7 (0.56)	18.8 (0.34)	<0.001
Acculturation level—SASH Language, mean (SE)^4^	2.1 (0.03)	2.2 (0.04)	2.1 (0.03)	0.0023
Chronic conditions, *n* (%)				
Hypertension	6048 (39.9)	1620 (43.0)	4428 (39.0)	0.0068
Angina	351 (2.0)	106 (2.9)	245 (1.8)	0.0143
Heart Failure	248 (1.6)	72 (1.9)	176 (1.5)	0.2236
Peripheral arterial disease	710 (4.5)	207 (5.3)	503 (4.3)	0.0946
Diabetes	2806 (16.2)	760 (17.7)	2046 (15.7)	0.0358
Chronic kidney disease	1494 (10.6)	350 (9.3)	1144 (11.0)	0.025
Liver problems (e.g., Hepatitis, cirrhosis)	912 (6.5)	257 (7.1)	655 (6.3)	0.2891
Cancer	538 (3.7)	182 (5.4)	356 (3.1)	<0.001
Number of chronic conditions, *n* (%)				<0.001
None	3992 (37.3)	841 (30.0)	3151 (39.5)	
1	4744 (34.6)	1288 (37.1)	3456 (33.9)	
≥2	4604 (28.1)	1330 (32.9)	3274 (26.6)	
BMI, *n* (%)				<0.001
Underweight (<18.5)	95 (1.2)	30 (1.3)	65 (1.1)	
Normal (18.5–24.9)	2556 (21.2)	730 (23.1)	1826 (20.6)	
Overweight (25.0–29.9)	5010 (37.6)	1376 (40.7)	3634 (36.6)	
Obese (30+)	5646 (40.0)	1317 (34.8)	4329 (41.7)	
Current cigarette smoker, *n* (%)				<0.001
No (i.e., never or former smoker)	10,887 (80.1)	2960 (84.6)	7927 (78.7)	
Yes (i.e., current smoker)	2431 (19.9)	493 (15.4)	1938 (21.3)	
Physical activity level (min/wk), *n* (%)				0.0093
High	1379 (12.7)	351 (11.6)	1028 (13.0)	
Moderate	5834 (44.5)	1597 (48.0)	4237 (43.4)	
Low	6081 (42.8)	1501 (40.3)	4580 (43.5)	
Alternate Healthy Eating Index-2010, mean (SE)^5^	47.6 (0.19)	49.5 (0.24)	47.1 (0.19)	<0.001

Abbreviations: GED, general education development; SASH, Short Acculturation Scale for Hispanics.

1 Frequencies provided are unweighted.

2 Percents, means, and *P* value analyses were weighted to account for complex sampling and study design of the Hispanic Community Health Study/Study of Latinos.

3 Frequencies provided do not add up to the total to account for not reported/ missing.

4 SASH Language score was determined by taking the mean of 4 items from the Short Acculturation Scale for Hispanics, which assessed language use in reading, speaking at home, thinking, and speaking with friends. Responses range from 1 to 5 with higher scores indicating better English language orientation.

5 The AHEI-2010 consists of 11 components—whole grains, fruit, vegetables, nuts and legumes, omega-3 fatty acids, polyunsaturated fatty acids, sugar-sweetened beverages and fruit juice, red and processed meats, trans fats, sodium, and alcohol—scored from 0 to 10 based on intake. Total scores range from 0 to 110, with higher scores indicating better dietary quality.

### Total usual dietary vitamin D intake

On mean, dietary supplement users had a total daily intake of 17.41 μg/d, whereas nonusers had an intake of 5.06 (Table 2). Among dietary supplement users, Puerto Rican individuals had the highest total daily intake (29.29), whereas Dominican individuals had the lowest (13.92). Among nonusers, Mexican (5.41), Cuban (5.30), and South American (5.28) individuals had higher total daily intakes, whereas Puerto Rican (4.98), Central American (4.70), and Dominican individuals had lower intakes (3.89). For more information on mean daily intakes, please see Supplemental Table 2.

**TABLE 2:** Mean daily total usual vitamin D intake in μg/d, from food intake, by dietary supplementation use and heritage, Hispanic Community Health Study/Study of Latinos, 2008–2011.

Heritage	Supplement users (μg)				Nonsupplement users (μg)			
	*n*	Mean (P25, P75)^1^	SE (Mean)	T-test *P* value^2^	*n*	Mean (P25, P75)	SE (Mean)	T-test *P* value
Overall	3459	17.41 (8.62, 17.46)	1.40		9881	5.06 (3.19, 6.43)	0.06	
Central American	357	14.60 (8.52, 17.14)	0.87	0.673	1066	4.74 (2.97, 6.02)	0.12	<0.001
Cuban	384	14.94 (9.51, 18.29)	0.43	0.882	1680	5.30 (3.41, 6.69)	0.10	0.245
Dominican	283	13.92 (7.40, 15.49)	0.93	0.299	899	3.89 (2.39, 4.97)	0.12	<0.001
Mexican	1457	15.04 (8.22, 17.21)	0.49	(ref)	3785	5.41 (3.48, 6.84)	0.07	(ref)
Puerto Rican	629	29.29 (9.75, 18.99)	7.69	0.063	1501	4.80 (3.03, 6.10)	0.12	<0.001
South American	248	17.19 (8.24, 16.03)	2.73	0.374	649	5.28 (3.39, 6.68)	0.15	0.346
Other/missing	101	14.88 (8.01, 17.18)	1.36	0.913	301	4.65 (2.89, 5.91)	0.23	<0.001

1 P25 indicates the 25^th^ percentile and P75 the 75^th^ percentile.

2 Means and *P* value analyses were weighted to account for complex sampling and study design of the Hispanic Community Health Study/Study of Latinos.

### EAR

**FIGURE 1:**
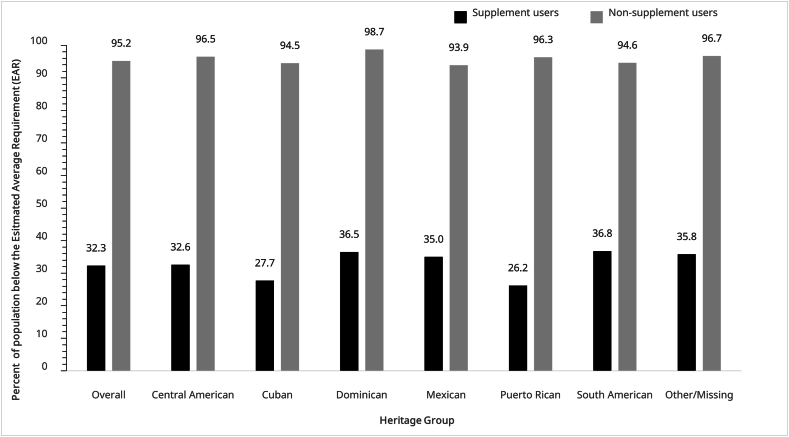
Prevalence below recommended dietary reference levels (EAR <10 mg/d or 400 International Units) for vitamin D by dietary supplementation use and heritage, Hispanic Community Health Study/Study of Latinos, 2008–2011.

Overall, dietary supplement users had significantly lower proportions below the EAR intake (32.3% compared with 95.2%) than nonusers (Supplemental Table 3 and Figure 1). Among nonusers, >90% of every heritage group were below the EAR. However, among dietary supplement users, Cuban (27.7%) and Puerto Rican individuals (26.2%) had the lowest proportion of individuals below the EAR.

### Mean contributions of dietary supplements

Overall, vitamin D usual supplement intake (12.27 μg/d) contributed a larger proportion (56.9%) to total usual intake (17.41) than usual food intake alone (5.14) (Table 3 and Supplemental Table 4). The relative contributions of supplements to total usual intake varied by heritage, with the highest contributions being among Dominican (61.9%), Puerto Rican (61.7%), and Central American individuals (59.7%). For usual food intake alone, Dominican (3.89), Central American (4.70), and Puerto Rican individuals (4.98) had the lowest mean intakes. With the addition of dietary supplements, Puerto Rican individuals had the highest mean intake (29.29) compared with all other heritages.

**TABLE 3:** Mean daily total usual vitamin D by food and dietary supplement intake and heritage among dietary supplement users only, Hispanic Community Health Study/Study of Latinos, 2008–2011.

		Supplement intake alone			Food intake alone			Food + supplements					
Heritage	*n*	Mean (P25, P75)^1^	SE (Mean)	T-test *P* value^2^	Mean (P25, P75)	SE (Mean)	T-test *P* value	Mean (P25, P75)	SE (Mean)	T-test *P* value	Mean Percent Contribution from Dietary Supplementation	SE (Mean)	T-test *P* value
Overall	3459	12.27 (3.33, 10.00)	1.10		5.14 (3.26, 6.52)	0.06		17.41 (8.62, 17.46)	1.40		56.86	5.65	
Central American	357	9.90 (4.00, 10.00)	0.70	0.6786	4.70 (2.94, 6.00)	0.12	<0.001	14.60 (8.52, 17.14)	0.87	0.673	59.73	1.22	<0.001
Cuban	384	9.57 (4.67, 12.50)	0.40	0.9959	5.36 (3.46, 6.78)	0.11	0.414	14.94 (9.51, 18.29)	0.43	0.882	57.83	1.01	<0.001
Dominican	283	10.03 (3.33, 10.00)	0.87	0.6250	3.89 (2.36, 4.96)	0.12	<0.001	13.92 (7.40, 15.49)	0.93	0.299	61.91	1.90	<0.001
Mexican	1457	9.57 (2.67, 10.00)	0.36	(ref)	5.47 (3.54, 6.89)	0.09	(ref)	15.04 (8.22, 17.21)	0.49	(ref)	53.07	0.92	(ref)
Puerto Rican	629	24.30 (5.00, 12.50)	6.15	0.0172	4.98 (3.15, 6.34)	0.13	<0.001	29.29 (9.75, 18.99)	7.69	0.063	61.73	1.09	<0.001
South American	248	11.89 (2.92, 10.00)	1.77	0.2004	5.30 (3.42, 6.69)	0.14	0.282	17.19 (8.24, 16.03)	2.73	0.374	53.71	1.64	0.720
Other/Missing	101	9.88 (3.33, 10.00)	1.40	0.8308	5.00 (3.26, 6.36)	0.25	0.071	14.88 (8.01, 17.18)	1.36	0.913	56.32	2.38	0.208

1 P25 indicates the 25^th^ percentile and P75 the 75^th^ percentile.

2 Means and *P* value analyses were weighted to account for complex sampling and study design of the Hispanic Community Health Study/Study of Latinos.

### Dietary food sources of vitamin D (servings/day)

Food source intake serving size definitions are reported above in the methods section and in Supplemental Table 5A and B. On mean, the most consumed dietary sources of vitamin D in the overall sample were dairy milk (0.699 serving/d), fish (0.655 serving/d), cheese (0.492 serving/d), eggs or egg substitutes (0.434 serving/d), margarine (0.390 serving/d), and citrus juice (0.339 serving/d). Among heritage groups, for dairy milk, Cubans (0.825 serving/d) had higher consumption, and Dominicans (0.494 serving/d) had lower consumption. For fish, South Americans (0.901 serving/d) had the highest consumption, and Puerto Ricans had the lowest consumption (0.508 serving/d). For citrus juice, South Americans (0.499 serving/d) had the highest consumption, and Mexicans had the lowest (0.307 serving/d).

## Discussion

Our findings showed that most United States Hispanic/Latino adults in the HCHS/SOL did not meet the daily recommended intake for vitamin D through food sources alone. Dietary supplement users were less likely to be below the recommended EAR than nonusers, but a substantial proportion still did not meet the EAR. Overall, our findings demonstrated inadequate vitamin D intake among Hispanic/Latino heritage groups, which is consistent with an earlier HCHS/SOL analysis that examined usual vitamin D intake among other nutrients [17]. Unlike prior studies from HCHS/SOL, our findings highlight significant differences in vitamin D intake among Hispanic/Latino adults by heritage. We found that Dominican, Central American, and Puerto Rican individuals had the lowest intake of vitamin D from food sources alone. Use of vitamin D supplements also varied by heritage group, with Puerto Rican individuals being the most likely to use supplements, and thus the most likely to meet EAR, and Dominican individuals were the least likely to use supplements. These findings underscore the potential role of dietary supplements in helping Hispanic/Latino adults achieve recommended intake concentrations, which may reduce the prevalence of vitamin D deficiency within this population.

Some individuals in our study met the dietary reference intake concentrations for vitamin D from dietary supplement use alone, similar to another study using NHANES data to estimate total daily usual vitamin D intake [29]. However, ∼50% of the United States population takes dietary supplements [30]. Our findings suggested that a larger proportion of Hispanic/Latino adults are not taking vitamin D supplements (74.1% nonusers of dietary supplement) and may benefit from supplementation. Our findings are consistent with a United States study among adults in a health plan population, which found that Latinos with inadequate vitamin D from food were less likely to make up the difference through dietary supplementation [31].

When assessing dietary supplements, there were no statistically significant differences in the amounts of vitamin D consumption among the heritage groups. Puerto Rican, Dominican, and Central American adults had the highest mean percent contribution of dietary supplements toward the total usual vitamin D intake. Therefore, our findings support the need for delivering culturally sensitive nutrition education applicable to all Hispanic/Latino individuals that can be further tailored by heritage [32]. Although vitamin D supplements are generally safe in modest doses, there are potential harms when very large doses are consumed without medical guidance. Greater emphasis is needed on the benefits of consuming various vitamin D-rich foods and adhering to recommended doses of dietary vitamin D supplements.

HCHS/SOL offers the opportunity to examine variation in dietary patterns across Hispanic/Latino heritage groups and whether these affect the adequacy of vitamin D consumption. For example, another HCHS/SOL study examining heritage-specific dietary patterns, found high levels of fish consumption among Cuban, Dominican, Mexican, and South American groups and high consumption of egg and cheese among Dominican, Puerto Rican, and Central American groups [33]. Although dietary patterns for Dominican, Central American, and Puerto Rican adults in our study were consistent with this other study, on mean, no heritage group met the EAR for vitamin D intake from food alone. In our study, the main vitamin D food sources consumed were dairy milk, fish, margarine, cheese, eggs/egg substitutes and citrus juice. Overall, for all the vitamin D food sources, mean servings were less than the Dietary Guidelines for Americans’ recommendations [34]. Although fatty fish naturally contains the largest amount of vitamin D, it is more expensive than other fish (e.g., tilapia) and protein sources, such as poultry, eggs, and beans [35]. For Hispanic/Latino adults in our study, this may be a barrier because the majority had lower household incomes.

Furthermore, the mean language acculturation score was low in our sample, which may lead to linguistic barriers and lower nutrition literacy, particularly in interpreting serving sizes and nutrition labels, as observed in United States Latino adult populations, potentially hindering their ability to meet dietary vitamin D recommendations from food sources alone [36,37]. United States Latino immigrants disproportionately experience factors such as lower educational attainment, higher food insecurity, and lack of health care access, leading to barriers to healthy eating [38]. Dietary supplements may be a costly expense for many low-income individuals, and could be reasonable to provide through food assistance programs (e.g., SNAP) [39]. A qualitative study in Canada identified barriers to dietary supplement use among low-income individuals, including income and accessibility [40]. In our study, nondietary supplement users were more likely to have educational attainment of less than high school, an income between $10,001 and $20,000, and no health insurance. Similarly, another study evaluating the role of health insurance coverage found that overall use of nutritional supplements increased after enrollment in a health plan [41]. They found that individuals with access to health insurance were more likely than those with no insurance to receive nutritional counseling through their primary care physicians and prescription vitamins/dietary supplements free of charge [41]. However, more studies are needed to investigate barriers to supplement use among Hispanic/Latino populations, especially because use varies across heritage groups.

The major limitation of this study is the lack of available vitamin D biomarker data to compare with self-reported dietary intake. However, we combined 2 24-h dietary recalls and the FPQ to reduce the measurement error intrinsic to self-reported dietary intake. The FPQ served as a second dietary recall source to collect dietary intake data on foods that are episodically consumed. The FPQ was administered one year after the time of the food frequency data collection, and although unlikely, nutritional patterns may have changed. Furthermore, we did not examine the regional impact on dietary patterns, however another study using HCHS/SOL data found that individuals of Mexican background from San Diego and Chicago sites shared similar dietary patterns, related to most of the selected variables: younger, first generation immigrants, who have lived <10 y in the United States, married/living with a partner, reporting an income <$30,000, and having less than a high school education [42]. This suggests that regardless of region, dietary patterns were more closely determined by socioeconomic status and heritage [42]. More research is needed to investigate regional impact among Hispanic/Latino heritage groups. In line with the scope of this paper, we adhered to the methodology described in *Best Practices for Dietary Supplement Assessment and Estimation of Total Usual Nutrient Intakes in Population-Level Research and Monitoring,* which examines one dietary component at a time [17]. We did not look at food source intakes stratified by supplement use due to the added complexity introduced by accounting for dietary supplements and the lack of widely accepted statistical methods. HCHS/SOL is not a nationally representative sample of all Hispanic/Latino adults in the United States; however, the 4 field centers represented in this study are among the United States cities with the largest Hispanic/Latino concentrations. This study has notable strengths, including dietary data collection methods similar to NHANES and NCI models, which have been applied to examine total usual intakes adjusting for the effects of random measurement error.

Overall, our findings suggest a need to promote the use of dietary supplementation of vitamin D, in addition to recommending foods high in vitamin D, to assist Hispanic/Latino adults at risk of not meeting dietary reference intakes (e.g., EAR). Furthermore, our study uncovered differences in the use of vitamin D dietary supplements and food sources by heritage. More research is needed on the impact of nutrition literacy, economic factors, the incorporation of biomarker data, regional impact on dietary patterns (northern compared with southern regions), and vitamin D fortification of foods among Hispanic heritage groups. There is also a need for improvement in culturally sensitive nutrition education to increase awareness on vitamin D deficiency, the use of dietary supplements, and consumption of vitamin D food sources according to the current Dietary Guidelines for Americans for all Hispanic/Latino heritages. Lastly, increasing access to personalized nutrition assessment and counseling to provide dietary guidance to Hispanic/Latino adults is needed. Nutritional counseling carried out by dietitians, nutritionists, and other healthcare professionals is recognized as the first-line approach in the management of nutritional status and prevention of numerous chronic diseases [43].

## Author contributions

The authors’ responsibilities were as follows – I. M., A. M. N., E. J. P.-S.: study conception and design; I. M., A. H.: data management; I. M., N. F: created the first draft of the manuscript; all authors: commented on previous versions of the manuscript; and all authors: read and approved the final manuscript.

## Disclaimer

This research was supported [in part] by the Intramural Research Program of the National Institutes of Health (NIH). The contributions of the NIH author(s) were made as part of their official duties as NIH federal employees, are in compliance with agency policy requirements, and are considered Works of the United States Government. However, the findings and conclusions presented in this paper are those of the author(s) and do not necessarily reflect the views of the NIH or the US Department of Health and Human Services.

## Data availability

All HCHS/SOL data are available for analysis by submitting a manuscript proposal to the HCHS/SOL Publications Committee at https://sites.cscc.unc.edu/hchs/.

## Funding

This work was financially supported by the Division of Intramural Research at the National Heart, Lung, and Blood Institute. The Hispanic Community Health Study/Study of Latinos is carried out as a collaborative study supported by contracts from the National Heart, Lung, and Blood Institute (NHLBI) to the University of North Carolina, Grant/Award Number: N01HC65233; University of Miami, Grant Number/Award Number: N01HC65234; Albert Einstein College of Medicine, Grant Number/Award Number: N01HC65235; Northwestern University, Grant Number/Award Number: N01HC65236; San Diego State University, Grant Number/Award Number: N01HC65237. The following institutes/centers/offices contribute to the HCHS/SOL through transfer of funds to the NHLBI: National Institute on Minority Health and Health Disparities, National Institute on Deafness and Other Communication Disorders, National Institute of Dental and Craniofacial Research, National Institute of Diabetes and Digestive and Kidney Diseases, National Institute of Neurological Disorders and Stroke, and Office of Dietary Supplements, all at the National Institutes of Health.

## Conflict of interest

The authors report no conflicts of interest.
