# Supplementary material for: Vitamin D Intake from Foods, Supplements, and Food Sources: Findings from the Hispanic Community Health Study/Study of Latinos
Source: J Nutr. 2025 Sep 10;155(11):3737–46. doi: 10.1016/j.tjnut.2025.09.002 (PMC12680955; doi:10.1016/j.tjnut.2025.09.002)
Supplement: Supplementary file 1 [file mmc1.docx]

**Supplemental Table 1:** Datasets and variables used for food sources

**For 24-hour food sources taken from dataset: dtsa_inv4.sas7bdat**

| **Food sources** | **Variables** |
| --- | --- |
| **Natural sources:** |  |
| Fish | DTSA69: MFF0100 Fish - Fresh and Smoked  DTSA70: MFL0100 Lean Fish - Fresh and Smoked  DTSA71:MFF0200 Fried Fish - Commercial Entrée and Fast Food  DTSA72:MSL0100 Shellfish  DTSA73: MSF0100 Fried Shellfish - Commercial Entrée and Fast Food |
| Organ Meats | /*DTSA76:MOF0100 Organ Meats*/ |
| Egg/egg substitute: | /*DTSA79:MOF0300 Eggs  DTSA80:MOF0400 Egg Substitute*/ |
| Cheese | /*DTSA95:DCF0100 Cheese - Full Fat  DTSA96:DCR0100 Cheese - Reduced Fat  DTSA97: DCL0100 Cheese - Low Fat and Fat Free  DTSA98: DCN0100 Cheese - Nondairy*/ |
| **Fortified Sources:** |  |
| Dairy (Milk) | /*DTSA84:DMF0100 Milk - Whole  DTSA85:DMR0100 Milk - Reduced Fat  DTSA86:DML0100 Milk - Low Fat and Fat Free  DTSA88:DMF0200 Ready-to-drink Flavored Milk - Whole  DTSA89:DMR0200 Ready-to-drink Flavored Milk - Reduced Fat  DTSA90:DML0200 Ready-to-drink Flavored Milk - Low Fat and Fat Free  DTSA91:DML0300 Sweetened Flavored Milk Beverage Powder with Non-fat Dry Milk  DTSA92: DML0400 Artificially Sweetened Flavored Milk Beverage Powder with Non-fat Dry Milk  DTSA93:SWT0600 Sweetened Flavored Milk Beverage Powder without Non-fat Dry Milk  DTSA94: MSC1100 Artificially Sweetened Flavored Milk Beverage Powder without Non-fat Dry Milk |
| Plant-based beverage | DTSA87:DMN0100 Milk - Nondairy*/ |
| Ready to eat cereal: | /*DTSA37:GRW0600 Ready-to-eat Cereal (not presweetened) - Whole Grain  DTSA38:GRS0600 Ready-to-eat Cereal (not presweetened) - Some Whole Grain  DTSA39:GRR0600 Ready-to-eat Cereal (not presweetened) - Refined Grain  DTSA40:RW0700 Ready-to-eat Cereal (presweetened) - Whole Grain  DTSA41:GRS0700 Ready-to-eat Cereal (presweetened) - Some Whole Grain  DTSA42:GRR0700 Ready-to-eat Cereal (presweetened) - Refined Grain*/ |
| Citrus juice: | /*DTSA4:FRU0100 Citrus Juice*/ |
| Yogurt | /*DTSA99:DYF0100 Yogurt - Sweetened Whole Milk  DTSA100:DYR0100 Yogurt - Sweetened Low Fat  DTSA101:DYL0100 Yogurt - Sweetened Fat Free  DTSA102:DYF0200 Yogurt - Artificially Sweetened Whole Milk  DTSA103:DYR0200 Yogurt - Artificially Sweetened Low Fat  DTSA104:DYL0200 Yogurt - Artificially Sweetened Fat Free  DTSA105:DYN0100 Yogurt - Nondairy*/ |
| Margarine | /*DTSA118:FMF0100 Margarine - Regular  DTSA119:FMR0100 Margarine - Reduced Fat*/ |

**FPQ: Food propensity questionnaire**

**Version A: dataset is called fpeA_afuinv9.sas7bdat**

| **Food source** | **Variable** |
| --- | --- |
| Citrus juice: | Variable: FPEA2  Label: Orange/grapefruit juice - how often |
| Milk | Frequency  Variable: FPEA7  Label: Milk as beverage - how often |
| Ready to eat cereal: | Variable: FPEA14  Label: Cold cereal - how often |
| Cheese | 1) FPEA110- cheese how often 2) FPEA109- cottage cheese how often |
| yogurt | PFEA108: yogurt how often |
| Eggs/eggs substitute: | 1) Variable: FPEA122  label :Eggs-how often  2) Variable: FPEA122A  label: Eggs-how often substitutes |
| Margarine | 1) FPEA132- Margarine-how often w/bread 2) FLEA132A, Margarine-how often low-fat w/breads 3) FPEA134 Margarine-how often on sides 4) FPEA134A,Margarine-how often low-fat on sides |
| Fish | 1) FPEA91-smoked fish  2) FPEA94-fish sticks  3) FPEA95-other fish |
| Organ meats | Not included |

**Version B: dataset is called: fpeb_afuinv9.sas7bdat**

| **Food source** | **Variables** |
| --- | --- |
| Citrus juice | FPEB1-drink orange juice |
| Milk | FPEB6-drink milk |
| Ready to eat cereal: | FPEB12-eat cold cereal |
| Eggs: | FPEB102-eat eggs, egg whites |
| Fish/fish products: | 1) variable FPEB78-eat smoked fish  2) variable FPEB79-eat other fish |
| Yogurt | FPEB88-Eat yogurt |
| Cheese | 1) FPEB89 eat cottage cheese 2) FPEB90-eat cheese |
| Margarine | Not included |
| Organ meats | Not included |

**Supplemental Table 2:** Mean daily total usual vitamin D intake in (IU)/day, from food intake, by dietary supplementation use and heritage, Hispanic Community Health Study/Study of Latinos, 2008-2011

|  | **Supplement users (**IU**)** | | |  | **Non-supplement users (**IU**)** | | |  |
| --- | --- | --- | --- | --- | --- | --- | --- | --- |
| **Heritage** | ***n*** | **Mean (P25, P75)^a^** | **SE (Mean)** | **T-test**  ***p*-value^b^** | ***n*** | **Mean (P25, P75)** | **SE (Mean)** | **T-test**  ***p*-value** |
| Overall | 3459 | 696.4 (344.8, 698.4) | 1.40 |  | 9881 | 202.4 (127.6, 257.2) | 0.06 |  |
| Central Amer. | 357 | 584.0 (340.8, 685.6) | 0.87 | 0.673 | 1066 | 189.6 (118.8, 240.8) | 0.12 | <0.001 |
| Cuban | 384 | 597.6 (380.4, 731.6) | 0.43 | 0.882 | 1680 | 212.0 (136.4, 267.6) | 0.10 | 0.245 |
| Dominican | 283 | 556.8 (296.0, 619.6) | 0.93 | 0.299 | 899 | 155.6 (95.6, 198.8) | 0.12 | <0.001 |
| Mexican | 1457 | 601.6 (328.8, 688.4) | 0.49 | (ref) | 3785 | 216.4 (139.2, 273.6) | 0.07 | (ref) |
| Puerto Rican | 629 | 1171.6 (390.0, 759.6) | 7.69 | 0.063 | 1501 | 192.0 (121.2, 244.0) | 0.12 | <0.001 |
| South Amer. | 248 | 687.6 (329.6, 641.2) | 2.73 | 0.374 | 649 | 211.2 (135.6, 267.2) | 0.15 | 0.346 |
| Other/Missing | 101 | 595.2 (320.4, 687.2) | 1.36 | 0.913 | 301 | 186.0 (115.6, 236.4) | 0.23 | <0.001 |

^a^P25 indicates the 25^th^ percentile and P75 the 75^th^ percentile.

^b^Means and p-value analyses were weighted to account for complex sampling and study design of the Hispanic Community Health Study/Study of Latinos.

**Supplemental Table 3:** Prevalence below recommended dietary reference levels (Estimated Average Requirement (EAR) <10 microgram/day or 400 International Units) for vitamin D by dietary supplementation use and heritage, Hispanic Community Health Study/Study of Latinos, 2008-2011

|  | **Supplement users (**μg**)** | | | | **Non-supplement users (**μg**)** | | | |
| --- | --- | --- | --- | --- | --- | --- | --- | --- |
| **Heritage** | ***n*** | **Pct below EAR** | **SE (%)** | **T-test**  ***p*-value** | ***n*** | **Pct below EAR** | **SE (%)** | **T-test**  ***p*-value** |
| Overall | 3459 | 32.3 | 0.90 |  | 9881 | 95.2 | 0.36 |  |
| Central Amer. | 357 | 32.6 | 2.87 | 0.464 | 1066 | 96.5 | 0.47 | <0.001 |
| Cuban | 384 | 27.7 | 1.93 | <0.001 | 1680 | 94.5 | 0.51 | 0.172 |
| Dominican | 283 | 36.5 | 3.69 | 0.721 | 899 | 98.7 | 0.23 | <0.001 |
| Mexican | 1457 | 35.0 | 1.38 | (ref) | 3785 | 93.9 | 0.46 | (ref) |
| Puerto Rican | 629 | 26.2 | 1.88 | <0.001 | 1501 | 96.3 | 0.48 | <0.001 |
| South Amer. | 248 | 36.8 | 2.95 | 0.588 | 649 | 94.6 | 0.73 | 0.294 |
| Other/Missing | 101 | 35.8 | 5.02 | 0.881 | 301 | 96.7 | 0.85 | <0.001 |

**Supplemental Table 4:**

Mean daily total usual vitamin D by food and dietary supplement intake in (IU)/day and heritage among dietary supplement users only, Hispanic Community Health Study/Study of Latinos, 2008-2011

|  | | **Supplement intake alone** | | | **Food intake alone** | | | **Food + supplements** | | |  |  |  |
| --- | --- | --- | --- | --- | --- | --- | --- | --- | --- | --- | --- | --- | --- |
| **Heritage** | ***n*** | **Mean**  **(P25, P75)^a^** | **SE (Mean)** | **T-test**  **P value^b^** | **Mean**  **(P25, P75)** | **SE (Mean)** | **T-test**  **P-value** | **Mean**  **(P25, P75)** | **SE (Mean)** | **T-test**  **P-value** | **Percent Contribution from Dietary Supplementation** | **SE (Mean)** | **T-test**  **P-value** |
| Overall | 3459 | 490.8 (133.2, 400.0) | 1.10 |  | 5.14 (3.26, 6.52) | 0.06 |  | 17.41 (8.62, 17.46) | 1.40 |  | 56.86 | 5.65 |  |
| Central Amer. | 357 | 396.0 (160.0, 400.0) | 0.70 | 0.6786 | 4.70 (2.94, 6.00) | 0.12 | <0.001 | 14.60 (8.52, 17.14) | 0.87 | 0.673 | 59.73 | 1.22 | <0.001 |
| Cuban | 384 | 382.8 (186.8, 500.0) | 0.40 | 0.9959 | 5.36 (3.46, 6.78) | 0.11 | 0.414 | 14.94 (9.51, 18.29) | 0.43 | 0.882 | 57.83 | 1.01 | <0.001 |
| Dominican | 283 | 401.2 (133.2, 400.0) | 0.87 | 0.6250 | 155.6 (94.4, 198.4) | 0.12 | <0.001 | 556.8 (296.0, 619.6) | 0.93 | 0.299 | 61.91 | 1.90 | <0.001 |
| Mexican | 1457 | 382.8 (106.8, 400.0) | 0.36 | (ref) | 218.8 (141.6, 275.6) | 0.09 | (ref) | 601.6 (328.8, 688.4) | 0.49 | (ref) | 53.07 | 0.92 | (ref) |
| Puerto Rican | 629 | 972.0 (200.0, 500.0) | 6.15 | 0.0172 | 199.2 (126.0, 253.6) | 0.13 | <0.001 | 1171.6 (390.0, 759.6) | 7.69 | 0.063 | 61.73 | 1.09 | <0.001 |
| South Amer. | 248 | 475.6 (116.8, 400.0) | 1.77 | 0.2004 | 212.0 (136.8, 267.6) | 0.14 | 0.282 | 687.6 (329.6, 641.2) | 2.73 | 0.374 | 53.71 | 1.64 | 0.720 |
| Other/Missing | 101 | 395.2 (133.2, 400.0) | 1.40 | 0.8308 | 200.0 (130.4, 254.4) | 0.25 | 0.071 | 595.2 (320.4, 687.2) | 1.36 | 0.913 | 56.32 | 2.38 | 0.208 |

^a^P25 indicates the 25^th^ percentile and P75 the 75^th^ percentile.

^b^ Means and p-value analyses were weighted to account for complex sampling and study design of the Hispanic Community Health Study/Study of Latinos.

**Supplemental Table 5A:** Mean daily total usual vitamin D food sources intake in servings/day using dietary assessments, by heritage, Hispanic Community Health Study/Study of Latinos, 2008-2011

|  | **Overall** | | | **Central Amer.** | | | | **Cuban** | | | | **Dominican** | | | |
| --- | --- | --- | --- | --- | --- | --- | --- | --- | --- | --- | --- | --- | --- | --- | --- |
|  | ***n*** | **Mean (P25, P75)^a^** | **SE (Mean)** | ***n*** | **Mean (P25, P75)** | **SE (Mean)** | **T-test**  **P value** | ***n*** | **Mean (P25, P75)** | **SE (Mean)** | **T-test**  **P value** | ***n*** | **Mean (P25, P75)** | **SE (Mean)** | **T-test**  **P value** |
| A. Fish | 13340 | 0.655 (0.282, 0.867) | 0.0234 | 1423 | 0.653 (0.286, 0.864) | 0.0490 | 0.426 | 2064 | 0.617 (0.270, 0.812) | 0.0311 | 0.054 | 1182 | 0.657 (0.298, 0.869) | 0.0578 | 0.521 |
| B. Eggs / eggs substitute | 13340 | 0.434 (0.193, 0.585) | 0.0097 | 1423 | 0.463 (0.220, 0.622) | 0.0243 | 0.033 | 2064 | 0.325 (0.140, 0.434) | 0.0159 | <0.001 | 1182 | 0.354 (0.157, 0.468) | 0.0232 | <0.001 |
| C. Milk, dairy | 13340 | 0.699 (0.311, 0.954) | 0.0132 | 1423 | 0.618 (0.271, 0.842) | 0.0297 | <0.001 | 2064 | 0.825 (0.388, 1.136) | 0.0269 | <0.001 | 1182 | 0.494 (0.193, 0.691) | 0.0270 | <0.001 |
| D. Milk, plant based*(NOCORR model) | 13340 | 0.048 (0.000, 0.000) | 0.0028 | 1423 | 0.053 (0.000, 0.000) | 0.0053 | 0.954 | 2064 | 0.050 (0.000, 0.000) | 0.0055 | 0.553 | 1182 | 0.032 (0.000, 0.000) | 0.0056 | <0.001 |
| E. Citrus Juice | 13340 | 0.339 (0.080, 0.416) | 0.0134 | 1423 | 0.343 (0.081, 0.421) | 0.0365 | 0.393 | 2064 | 0.311 (0.072, 0.378) | 0.0261 | 0.917 | 1182 | 0.406 (0.103, 0.505) | 0.0454 | 0.049 |
| F. RTE-Cereals | 13340 | 0.162 (0.011, 0.162) | 0.0084 | 1423 | 0.151 (0.011, 0.156) | 0.0150 | 0.010 | 2064 | 0.095 (0.006, 0.087) | 0.0089 | <0.001 | 1182 | 0.139 (0.010, 0.136) | 0.0226 | 0.012 |
| G. Organ meat | 13340 | 0.064 (0.028, 0.080) | 0.3245 | 1423 | 0.063 (0.033, 0.080) | 0.3360 | 0.686 | 2064 | 0.037 (0.020, 0.048) | 0.3242 | 0.028 | 1182 | 0.032 (0.016, 0.041) | 0.2216 | 0.656 |
| H. Yogurt | 13340 | 0.053 (0.002, 0.047) | 0.0029 | 1423 | 0.030 (0.001, 0.023) | 0.0033 | <0.001 | 2064 | 0.073 (0.004, 0.072) | 0.0058 | <0.001 | 1182 | 0.041 (0.002, 0.033) | 0.0076 | 0.086 |
| I. Margarine | 13340 | 0.390 (0.152, 0.510) | 0.0157 | 1423 | 0.443 (0.190, 0.584) | 0.0327 | <0.001 | 2064 | 0.580 (0.274, 0.762) | 0.0327 | <0.001 | 1182 | 0.360 (0.167, 0.467) | 0.0359 | <0.001 |
| J. Cheese | 13340 | 0.492 (0.279, 0.654) | 0.0109 | 1423 | 0.401 (0.223, 0.533) | 0.0174 | 0.010 | 2064 | 0.417 (0.220, 0.559) | 0.0146 | <0.001 | 1182 | 0.455 (0.256, 0.605) | 0.0224 | 0.012 |

Serving sizes have been assigned to each NDSR food based on the recommendations made by the 2000 Dietary Guidelines for Americans when available.

Fish: one serving is equal to 1 oz

Eggs/egg substitutes: one serving is equal to 1 large egg

Milk: one serving is equal to 1 cup fluid milk

Citrus juice: one serving is equal to 4 fluid ounces

Ready to eat cereal (RTE): one serving is 1 ounce of ready-to-eat cereal

^a^P25 indicates the 25th percentile and P75 the 75th percentile

^b^Means and p-value analyses were weighted to account for complex sampling and study design of the Hispanic Community Health Study/Study of Latinos.

**Supplemental Table 5B:** Mean daily total mean usual vitamin D food sources intake in servings/day using dietary assessments, by heritage, Hispanic Community Health Study/Study of Latinos, 2008-2011

|  | **Mexican** | | | | **Puerto Rican** | | | | **South Amer.** | | | | **Other Missing** | | | |
| --- | --- | --- | --- | --- | --- | --- | --- | --- | --- | --- | --- | --- | --- | --- | --- | --- |
|  | ***n*** | **Mean (P25, P75)^a^** | **SE (Mean)^b^** | **T-test**  **P value** | ***n*** | **Mean (P25, P75)** | **SE (Mean)** | **T-test**  **P value** | ***n*** | **Mean (P25, P75)** | **SE (Mean)** | **T-test**  **P value** | ***n*** | **Mean (P25, P75)** | **SE (Mean)** | **T-test**  **P value** |
| A. Fish | 5242 | 0.695 (0.302, 0.921) | 0.0312 | (ref) | 2130 | 0.508 (0.218, 0.668) | 0.0382 | <0.001 | 897 | 0.901 (0.422, 1.200) | 0.0677 | <0.001 | 402 | 0.732 (0.314, 0.969) | 0.1112 | 0.745 |
| B. Eggs / eggs substitute | 5242 | 0.522 (0.256, 0.701) | 0.0169 | (ref) | 2130 | 0.441 (0.204, 0.592) | 0.0187 | <0.001 | 897 | 0.355 (0.158, 0.473) | 0.0250 | <0.001 | 402 | 0.442 (0.195, 0.594) | 0.0464 | 0.096 |
| C. Milk, dairy | 5242 | 0.710 (0.323, 0.959) | 0.0176 | (ref) | 2130 | 0.680 (0.331, 0.912) | 0.0242 | 0.223 | 897 | 0.723 (0.339, 0.987) | 0.0347 | 0.735 | 402 | 0.598 (0.246, 0.839) | 0.0426 | 0.012 |
| D. Milk, plant based*(NOCORR model) | 5242 | 0.053 (0.000, 0.000) | 0.0038 | (ref) | 2130 | 0.036 (0.000, 0.000) | 0.0043 | <0.001 | 897 | 0.061 (0.000, 0.000) | 0.0058 | 0.176 | 402 | 0.050 (0.000, 0.000) | 0.0065 | 0.648 |
| E. Citrus Juice | 5242 | 0.307 (0.076, 0.385) | 0.0200 | (ref) | 2130 | 0.320 (0.072, 0.382) | 0.0320 | 0.722 | 897 | 0.499 (0.129, 0.623) | 0.0463 | <0.001 | 402 | 0.470 (0.125, 0.593) | 0.0724 | 0.027 |
| F. RTE-Cereals | 5242 | 0.200 (0.016, 0.214) | 0.0120 | (ref) | 2130 | 0.197 (0.016, 0.213) | 0.0212 | 0.905 | 897 | 0.109 (0.007, 0.100) | 0.0170 | <0.001 | 402 | 0.192 (0.015, 0.200) | 0.0376 | 0.846 |
| G. Organ meat | 5242 | 0.078 (0.040, 0.100) | 0.3163 | (ref) | 2130 | 0.061 (0.032, 0.078) | 0.3024 | 0.685 | 897 | 0.059 (0.031, 0.076) | 0.3329 | 0.684 | 402 | 0.166 (0.092, 0.210) | 0.7486 | 0.849 |
| H. Yogurt | 5242 | 0.055 (0.003, 0.053) | 0.0040 | (ref) | 2130 | 0.036 (0.001, 0.028) | 0.0044 | <0.001 | 897 | 0.058 (0.003, 0.055) | 0.0079 | 0.715 | 402 | 0.048 (0.002, 0.044) | 0.0120 | 0.551 |
| I. Margarine | 5242 | 0.227 (0.097, 0.295) | 0.0103 | (ref) | 2130 | 0.442 (0.206, 0.576) | 0.0325 | <0.001 | 897 | 0.552 (0.249, 0.724) | 0.0487 | <0.001 | 402 | 0.402 (0.173, 0.520) | 0.0506 | <0.001 |
| J. Cheese | 5242 | 0.556 (0.339, 0.727) | 0.0172 | (ref) | 2130 | 0.537 (0.313, 0.708) | 0.0209 | 0.905 | 897 | 0.437 (0.251, 0.575) | 0.0213 | <0.001 | 402 | 0.495 (0.298, 0.647) | 0.0449 | 0.846 |

Serving sizes have been assigned to each NDSR food based on the recommendations made by the 2000 Dietary Guidelines for Americans when available.

Fish: one serving is equal to 1 oz

Eggs/egg substitutes: one serving is equal to 1 large egg

Milk: one serving is equal to 1 cup fluid milk

Citrus juice: one serving is equal to 4 fluid ounces

Ready to eat cereal (RTE): one serving is 1 ounce of ready-to-eat cereal

^a^P25 indicates the 25th percentile and P75 the 75th percentile

^b^Means and p-value analyses were weighted to account for complex sampling and study design of the Hispanic Community Health Study/Study of Latinos.
